# WALTER: an easy way to online evaluate telomere lengths from terminal restriction fragment analysis

**DOI:** 10.1186/s12859-021-04064-0

**Published:** 2021-03-22

**Authors:** Martin Lyčka, Vratislav Peska, Martin Demko, Ioannis Spyroglou, Agata Kilar, Jiří Fajkus, Miloslava Fojtová

**Affiliations:** 1 Mendel Centre for Plant Genomics and Proteomics, Central European Institute of Technology (CEITEC), Masaryk University, 625 00 Brno, Czech Republic; 2 National Centre for Biomolecular Research, Faculty of Science, Masaryk University, 625 00 Brno, Czech Republic; 3 Institute of Biophysics, Academy of Sciences of the Czech Republic, v.v.i., 612 00 Brno, Czech Republic; 4 Core Facility Bioinformatics, Central European Institute of Technology (CEITEC), Masaryk University, 625 00 Brno, Czech Republic; 5 Faculty of Informatics, Masaryk University, 602 00 Brno, Czech Republic

**Keywords:** Telomere length, Terminal restriction fragments, Online toolset

## Abstract

**Background:**

Telomeres, nucleoprotein structures comprising short tandem repeats and delimiting the ends of linear eukaryotic chromosomes, play an important role in the maintenance of genome stability. Therefore, the determination of the length of telomeres is of high importance for many studies. Over the last years, new methods for the analysis of the length of telomeres have been developed, including those based on PCR or analysis of NGS data. Despite that, terminal restriction fragment (TRF) method remains the gold standard to this day. However, this method lacks universally accepted and precise tool capable to analyse and statistically evaluate TRF results.

**Results:**

To standardize the processing of TRF results, we have developed WALTER, an online toolset allowing rapid, reproducible, and user-friendly analysis including statistical evaluation of the data. Given its web-based nature, it provides an easily accessible way to analyse TRF data without any need to install additional software.

**Conclusions:**

WALTER represents a major upgrade from currently available tools for the image processing of TRF scans. This toolset enables a rapid, highly reproducible, and user-friendly evaluation of almost any TRF scan including in-house statistical evaluation of the data. WALTER platform together with user manual describing the evaluation of TRF scans in detail and presenting tips and troubleshooting, as well as test data to demo the software are available at https://www.ceitec.eu/chromatin-molecular-complexes-jiri-fajkus/rg51/tab?tabId=125#WALTER and the source code at https://github.com/mlyc93/WALTER.

## Background

Telomeres are nucleoprotein structures delimiting the ends of linear eukaryotic chromosomes. They play an undisputedly significant role in the maintenance of genome stability protecting chromosomal ends from degradation and distinguishing natural chromosome ends from double stranded DNA breaks. Disruption of telomere homeostasis thus results in chromosomal non-stability and activation of processes of DNA damage response. For that reason, determining the length of telomeres is of great importance in basic and applied research and in clinical practice. This can be demonstrated by more than 13,400 papers in the WoS database directly mentioning "telomere length" in a title, abstract or key words, amounting to over 1000 papers annually in recent years. Over the last few years, new methods for the analysis of telomere length have been developed, including those based on quantitative PCR or evaluation of NGS data [1]. Tools utilizing NGS data for the purpose of telomere length measurement were recently comprehensively reviewed [2]. In this respect, the terminal restriction fragment (TRF) method represents the "gold standard" for telomere length analysis providing valuable information about the distribution of absolute telomere lengths, thus revealing the level of heterogeneity in telomere lengths. Moreover, TRF is applicable for analysis of a broad range of telomeres with respect to their lengths, starting from less than 1 kb [3] to hundreds of kilobases [4]. TRF analysis is based on the digestion of genomic DNA by a frequently cutting restriction enzyme that does not recognize telomeric repeats. Intact telomeres are visualised by Southern hybridization using labelled telomeric probe [5]. Accomplishment of the TRF protocol is therefore relatively simple (although time-consuming and requiring a large amount of intact genomic DNA), but evaluation of telomere-specific signals represents the challenging part of the analysis. Several tools were developed for this purpose, namely Telometric [6] and TeloTool [7], with each having its advantages but ultimately suffering from drawbacks that still force researchers to present raw TRF scans. However, this introduces a subjective component to the evaluation of telomere-specific signals.

To overcome these problems and to enable the evaluation of TRF scans suffering from low resolution, staining and telomere-non-specific signals, we have developed WALTER (Web-based Analyser of the Length of Telomeres). WALTER is a readily accessible and freely available online toolset that consists of two independent tools, ScanToIntensity and IntensityAnalyser, and enables a rapid, reproducible, and user-friendly evaluation of almost any TRF scan.

## Implementation

**Fig. 1:**
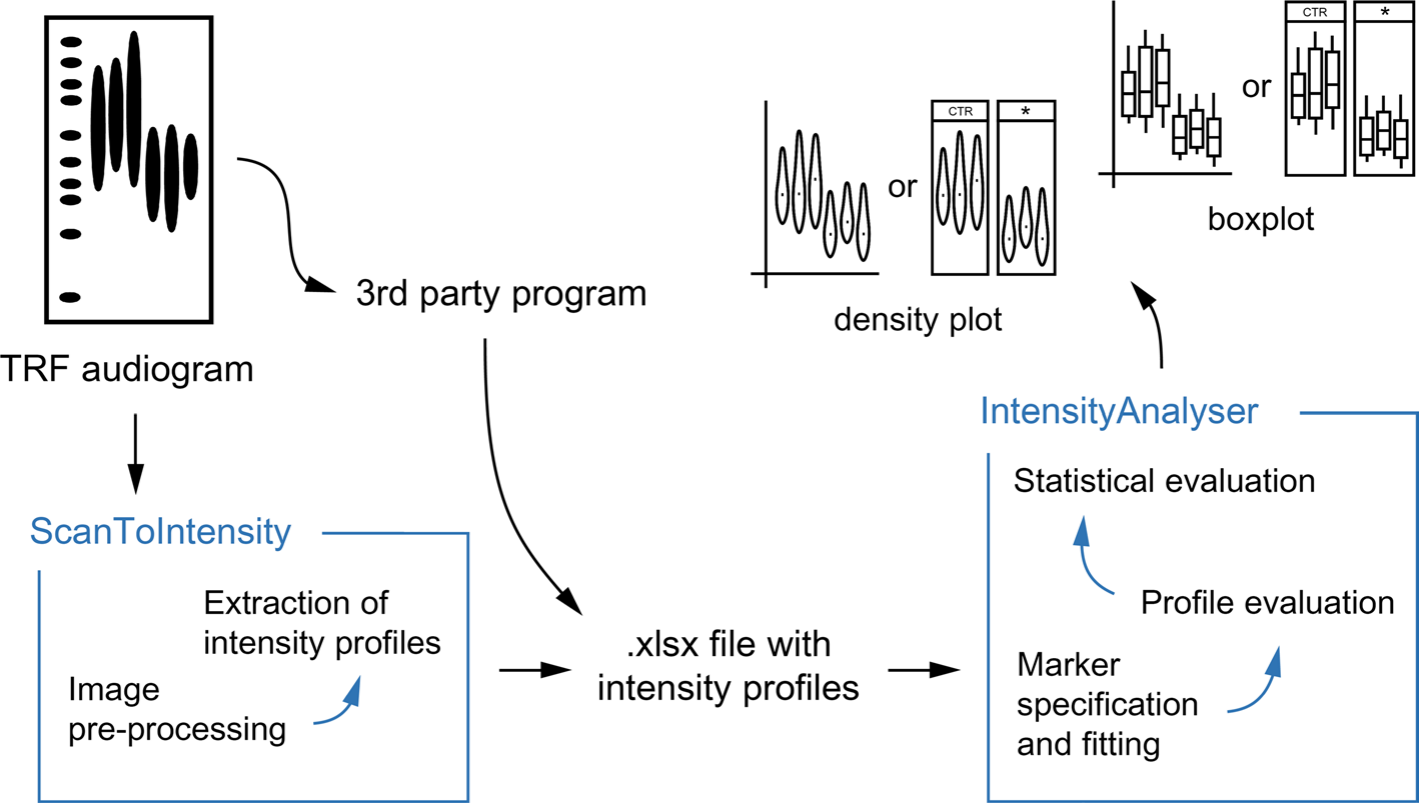
WALTER toolset workflow. The ScanToIntensity tool pre-processes the image and extracts intensity profiles and the IntensityAnalyser tool analyses those intensity profiles resulting in a plot output with possibility of statistical evaluation of the data. In case that TRF audiograms are curved and thus not eligible for the evaluation by the ScanToIntensity tool, 3rd party software is recommended for the transformation of the TRF scan into intensity profiles. (Figure created using Adobe Illustrator CS6)

The WALTER toolset was developed in R programming language using the Shiny package [8] and is running on a server administered by the Core Facility Bioinformatics, CEITEC Masaryk University, Brno, Czech Republic. The typical workflow of the evaluation of TRF scans by WALTER usually includes two steps: conversion of the telomere-specific signals to intensity profiles by the ScanToIntensity tool, and analysis of intensity profiles by the IntensityAnalyser tool. However, IntensityAnalyser can also process intensity profiles generated by the third-party programs, e.g., MultiGauge Ver. 3.0 (FujiFilm) or ImageJ (illustrated in Fig. 1).

### ScanToIntensity

The conversion of telomere-specific signals in TRF scans to intensity profiles is carried out by the ScanToIntensity tool. It accepts TRF scan in JPEG or BMP format (limit 100 MB). The uploaded image is automatically converted to grayscale so that each pixel intensity ranges from 0 to 1. It should be noted that for accurate analysis, a loading of DNA samples within the dynamic range of the signal intensity is required. The tool allows pre-processing of the image for better orientation and manipulation of its size as well as inverting colours for better visibility of sample lines. The intensity profile calculation is based on a summation of each row in a matrix computed from the selected area of the uploaded grayscale image. Given that selection of the area for the DNA size marker and each sample is in the hands of the user, the tool allows operators to highlight several DNA size markers in different adjacent lines and out of them, create just one intensity profile. The resulting .xlsx file is downloaded. This file, apart from the intensity profiles, also carries the information about the position of the selected area on the TRF scan used for the intensity profile calculation.

### IntensityAnalyser

This tool is used for the analysis of intensity profiles generated by the ScanToIntensity tool or any third-party program in .xls or .xlsx format.

#### DNA size marker specification and fitting

Analysis requires specification of the used DNA size marker. This can be done either manually by the user or chosen from pre-selected options. Next, the peak number corresponding to the position of the fragment of the respective length in the marker(s) intensity profile is assigned. For better clarity, the sensitivity of the peak search in the marker intensity profile is adjustable. A relationship between the length of the fragment and its distance in the gel (measured in pixels) is calculated. The result is automatically fitted with a polynomial function, the order of which is based on successive ANOVA hypothesis testing between two adjacent polynomial models. The null hypothesis is always that the simpler model is better than the more complex model. The model with the order of polynomial function that meets the criterion p ≤ 0.05 (null hypothesis rejected) last is used. Individual polynomial adjustment is also possible.

#### Marker correction

A correction for the uneven migration of DNA between distant samples is possible. In this case, the second marker is loaded on the opposite side of the gel. The correction requires a specification of the second marker in the same manner as done for the first marker. Linear models are then calculated between the fragments of the same length. These linear models are used to calculate virtual markers for each sample. In the case, that the second marker is not loaded to the furthest line, the linear model is extrapolated. For each virtual marker, a relationship between the length of the respective fragment and its distance in the gel is calculated with the result automatically fitted with the polynomial function. Each virtual marker is tested for the best order of the polynomial function to fit the data as mentioned above and the most frequent value is used for all virtual markers.

#### Selection of telomere-specific signal from the intensity profile

There are two ways how to select the part of the intensity profile that corresponds to the telomere-specific signal. Firstly, the area corresponding to the telomere smear is highlighted automatically.

The intensity values for the left (1) and right (2) borders of the highlighted area are calculated as follows:

1 $$OD_{lt} = OD_{lb} + {\text{ W}}\left( {OD_{{lmax{ }}} { } - { }OD_{lb} } \right)$$

2 $$OD_{rt} = OD_{rb} + {\text{W}}\left( {OD_{rmax} { } - { }OD_{rb} } \right)$$

where *OD*_*lmax*_ is the maximum signal intensity within the sequence $\left\{ {OD_{k} } \right\}_{k = 1}^{r}$, where *r* is the right pixel number specified for the first peak area; *OD*_*lb*_ is the lowest intensity value within the sequence $\left\{ {OD_{k} } \right\}_{k = 1}^{i}$, where *i* is the input of *OD*_*lmax*_; *OD*_*rmax*_ is the maximum signal intensity within the sequence $\left\{ {OD_{k} } \right\}_{k = l}^{n}$, where *n* is the number of pixels on the TRF scan and *l* is the left pixel number specified for the last peak area; *OD*_*rb*_ is the lowest intensity value within the sequence $\left\{ {OD_{k} } \right\}_{k = j}^{n}$, where *n* is the number of pixels on the TRF scan and *j* is the input of *OD*_*rmax*_; W is a constant influencing the width of the highlighted area with its value set by default to 0.28 based on empirical testing. The intensity profiles data for the border calculation are,* a priori*, smoothed with a smoothing span of 5%.

The area corresponding to the telomere signal is necessary to specify if the telomere-specific signal does not have a standard unimodal distribution, or if there are areas with a higher maximum intensity than in the telomere-specific signal. In such cases, the user highlights and saves the pixel position of the area corresponding to the first and last peak of the telomere-specific signal (the last peak specification might differ in TRF scans with a non-unimodal distribution of telomere-specific signal; for unimodal distribution, area for the first and last peak is the same).

The other option is to manually highlight and save the area of the telomere-specific signal in the TRF scan. However, the automatic selection allows effortless reproduction of the analysis and easy application of the background correction. Thus, automatic selection of the telomere-specific signals is unequivocally recommended.

#### Background correction

**Fig. 2:**
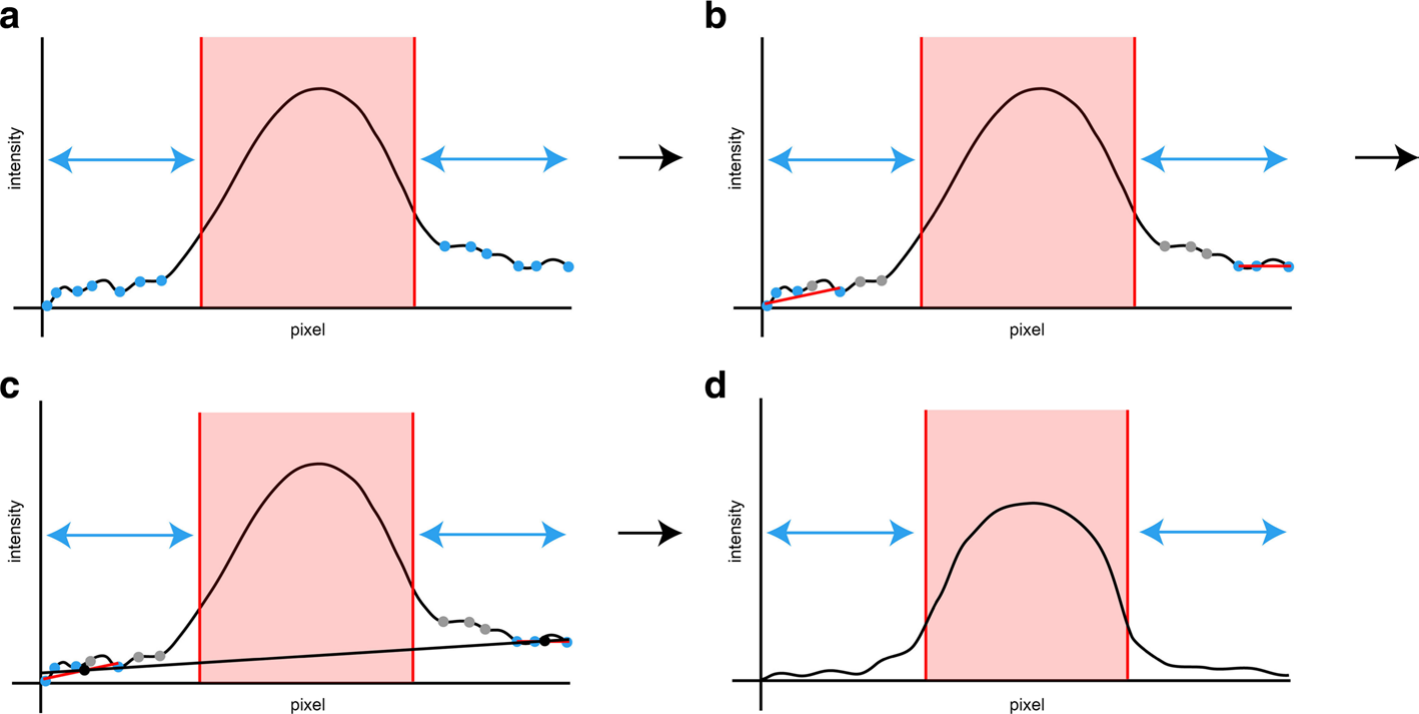
Calculation of background correction. (**a**) Finding local minima in intervals between the start (end) of the intensity profile and left (right) side of the selected area. (**b**) Calculation of the linear model on left and right side through points that have lower intensities than is the median of all points for that interval (at least 3 points needed). (**c**) Calculation of the linear model through the points that are in the middle of the linear models (if number of points < 3, minima of intervals are used instead). (**d**) Recalculation of the intensity profile by taking out the area under the linear model calculated in the previous panel C from the intensity profile (negative values are taken as zero). (Figure created using Adobe Illustrator CS6)

**Fig. 3:**
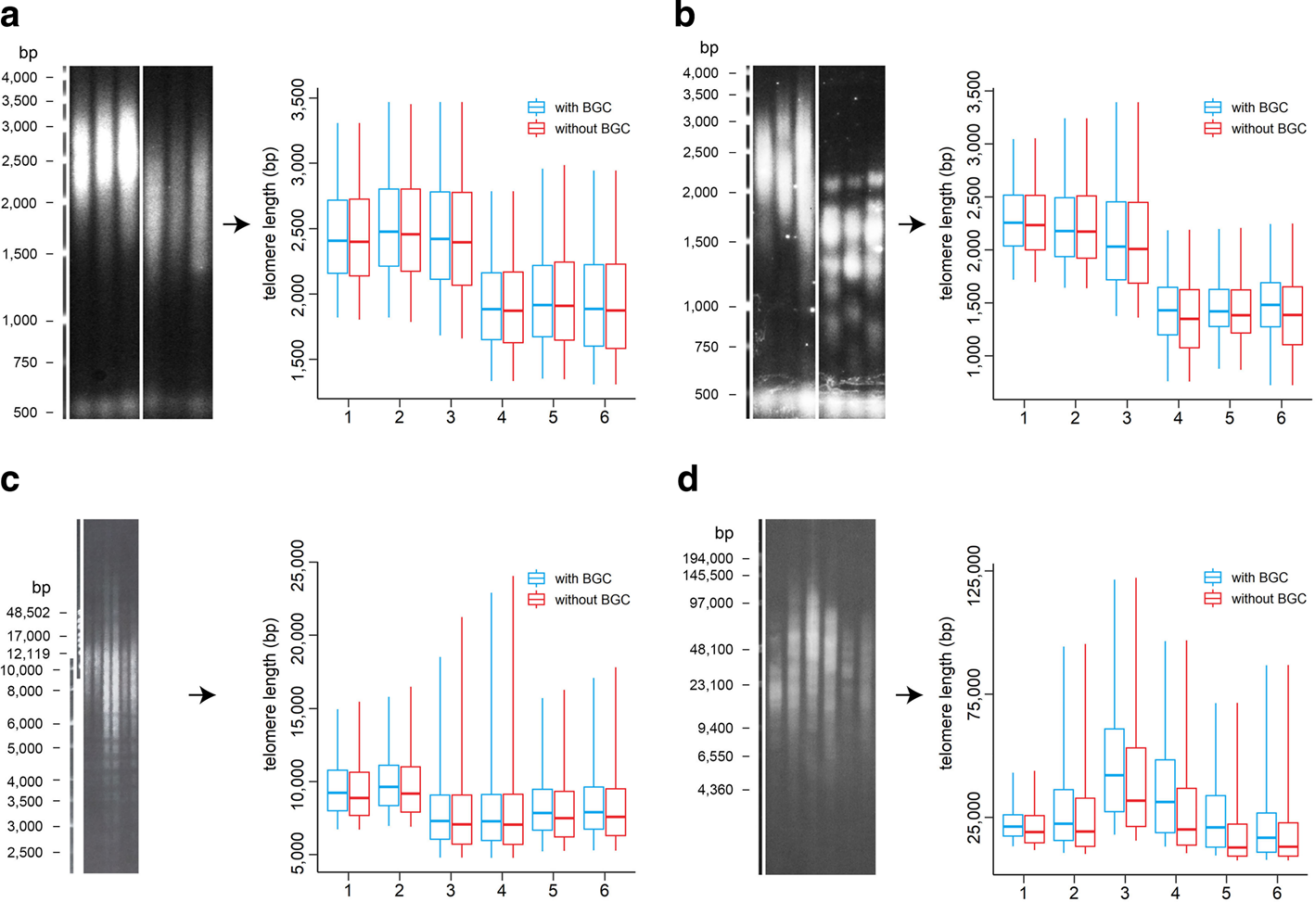
Influence of background correction on the WALTER result. Telomeres of *Arabidopsis thaliana* control and mutant plants **(a)**, *Arabidopsis thaliana* control plants and *tert* mutants (SALK_061434; loss of function of the gene encoding the telomerase protein subunit) with typical band-like pattern (**b**), human cells (**c**), and *Nicotiana* species (**d**) were analysed. In the boxplot, the central line indicates the weighted median, box limits indicate the 25th and 75th percentiles and whiskers indicate the minimum and maximum of the selected area of telomeres. BGC—background correction. (Figure created using Adobe Photoshop CS6 and R 3.5.3)

When the automatically selected area corresponds to the telomere-specific signal, correction of the background of the intensity profile prior to its evaluation is possible (Fig. 2). Given that the estimation of the weighted median and quartiles considers that the number of fragments with lower fragment size is higher than the number of fragments with higher fragment size with the same intensity, the absence of background correction will often lead to an underestimation of the telomere-specific signal. This is true especially for telomeres of relatively high lengths or if the background intensity of the TRF scan is not negligible and equally distributed (Fig. 3). The correction is based on a deduction of a linear model constructed from two points located on the left and right sides of the telomere-specific signal. The left side point is calculated from the interval of the intensity profile and consisted of the lowest pixel number up to the left border of the highlighted area. In this interval, all local minima that are lower than their median value are used for the construction of the linear model. The left side point then corresponds to the value in the middle of the linear model. Where there are just two or fewer points for the calculation of the linear model, the minimum of the interval is used instead as the left point. The right-side point is calculated in the same manner; however, in this case the range is from the right border of the highlighted area to the highest pixel number (Fig. 2).

#### Evaluation of telomere-specific signal

The weighted median of telomere length from the selected area of the telomere-specific signal is calculated for each sample using the sequence of the cumulative sum of weighted signal intensities $\left\{ {C_{k} } \right\}_{k = 1}^{n}$, where each value is defined as

$$C_{n} = \mathop \sum \limits_{k = 1}^{n} \left( {\frac{{OD_{k} }}{{L_{k} }}} \right) = \frac{{OD_{1} }}{{L_{1} }} + \cdots + \frac{{OD_{n} }}{{L_{n} }}$$

where *n* is the number of pixels of the TRF scan; *OD*_*i*_ is the signal intensity of pixel *i* within the sequence $\left\{ {OD_{k} } \right\}_{k = 1}^{n}$, where *n* is the number of pixels of the TRF scan; *L*_*i*_ is the fragment size of pixel *i* within the sequence $\left\{ {L_{k} } \right\}_{k = 1}^{n}$, where *n* is the number of pixels of the TRF scan.

The weighted median of telomere lengths is then defined as a predicted value of *L* (fragment size) for a given $\frac{{C_{n} }}{2}$ value of *C* (cumulative sum of weighted signal intensity) from a linear model based on two points $\left[ {C_{i} ;L_{i} } \right]$ and $\left[ {C_{i + 1} ;L_{i + 1} } \right]$, where *C*_*i*_ and *C*_*i*+1_ are values from the sequence $\left\{ {C_{k} } \right\}_{k = 1}^{n}$ defined as

$$C_{i} \le \frac{{C_{n} }}{2} < C_{i + 1} ;\quad i = 1,2, \ldots ,n - 1$$

and *L*_*i*_ and *L*_*i*+1_ are values of the same input (*i*, *i* + 1) from the sequence $\left\{ {L_{k} } \right\}_{k = 1}^{n}$. 1st and 3rd quartiles are based on the calculated weighted median of telomere length, while the minimum and maximum of the boxplot corresponds to the selected area of the telomere-specific signal. Before evaluation, data of intensity profiles are,* a priori*, smoothed with a smoothing span of 5%.

#### Presentation of results

The tool enables the presentation of results as boxplots or violin plots. A boxplot represents a conventional way of presenting a telomere-specific signal. However, a violin plot brings more complex information because it shows the distribution of the telomere-specific signal. This method of data portrayal is especially beneficial in cases of non-unimodal distribution of the telomere-specific signal. To ensure maximum reproducibility of the evaluation process, the tool produces a report with details of the analysis.

#### Recalculation of the data prior to statistical evaluation

Outcomes of the evaluation of telomere lengths are recalculated to means and standard deviations (SD) according to Wan et al. [9] using information about their interquartile range and weighted median with an adjustment where the number of samples (n) is considered as number of pixels in the whole range where the boxplot/violin plot is calculated. The calculated mean, SD and n of each sample is then used to combine samples according to their group name using formulas specified in the Cochrane Handbook for Systematic Reviews of Interventions [10]. The resulting mean and SD for each group is used to create mock data with these parameters (n here is considered as the number of samples in each group) for the statistical evaluation.

#### Statistical analysis of the results

The WALTER toolset facilitates a statistical evaluation of the significance of differences in telomere lengths among groups where this is frequently an ultimate goal of the analysis. All groups must consist of at least three samples. However, having five or more samples per group is strongly recommended to increase the statistical power of the test and to avoid possible false negative results. Comparison of two groups is always done by Welch's t-test. In cases of 3 or more groups, data can be compared by multiple Welch's t-test either against the control group or against each other. However, in those tests, the p-value is not adjusted for multiple testing. Stringent testing can be achieved with the Tamhane-Dunnett test that compares case groups to the control group or by the Games-Howell test that compares groups between each other. Given that type II error is considered as more plausible, it is recommended to use the multiple Welch's t-test. For the same reason, significance is set as: *< 0.1, **< 0.05, ***< 0.01 p-value. All tests are performed as two-tailed.

**Fig. 4:**
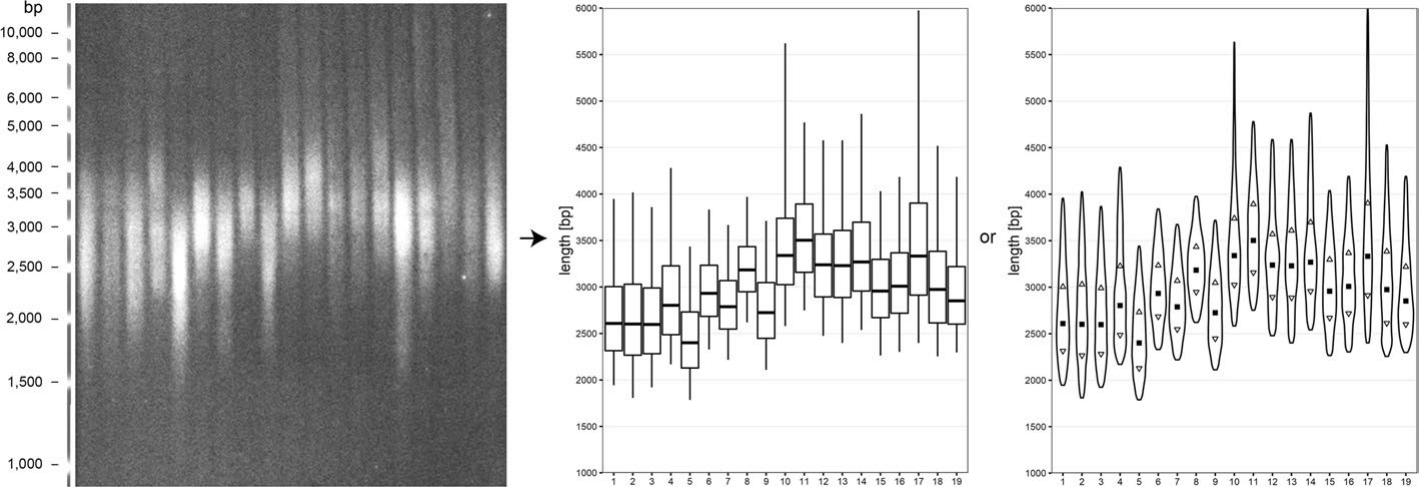
Inter-individual variability in telomere lengths in *Arabidopsis thaliana* plants. TRF scan of 19 samples of *Arabidopsis thaliana* ecotype *Columbia* and plots from WALTER analysis. In the boxplot, the central line indicates the weighted median, box limits indicate the 25th and 75th percentiles and whiskers indicate the minimum and maximum of the selected area of telomeres. In the violin plot, the rectangle indicates the weighted median, triangles indicate the 25th and 75th percentiles and the shape of the violin plot depicts the intensity changes between the minimum and maximum of the selected area of telomeres. Variance of the weighted medians of telomere lengths is approximately 1100 bp. (Figure created using Adobe Photoshop CS6)

At first glance, the statistical evaluation appears to be relatively insensitive, especially in cases of a small number of samples per group. However, telomeres do have a relatively high inter-individual variation in lengths that needs to be considered. For example, based on the analysis of 19 samples of *Arabidopsis thaliana* plants of the Columbia ecotype, the calculated weighted medians of telomere length varied around 1100 bp (Fig. 4).

## Results

### Influence of TRF scan properties on the WALTER outcome

**Fig. 5:**
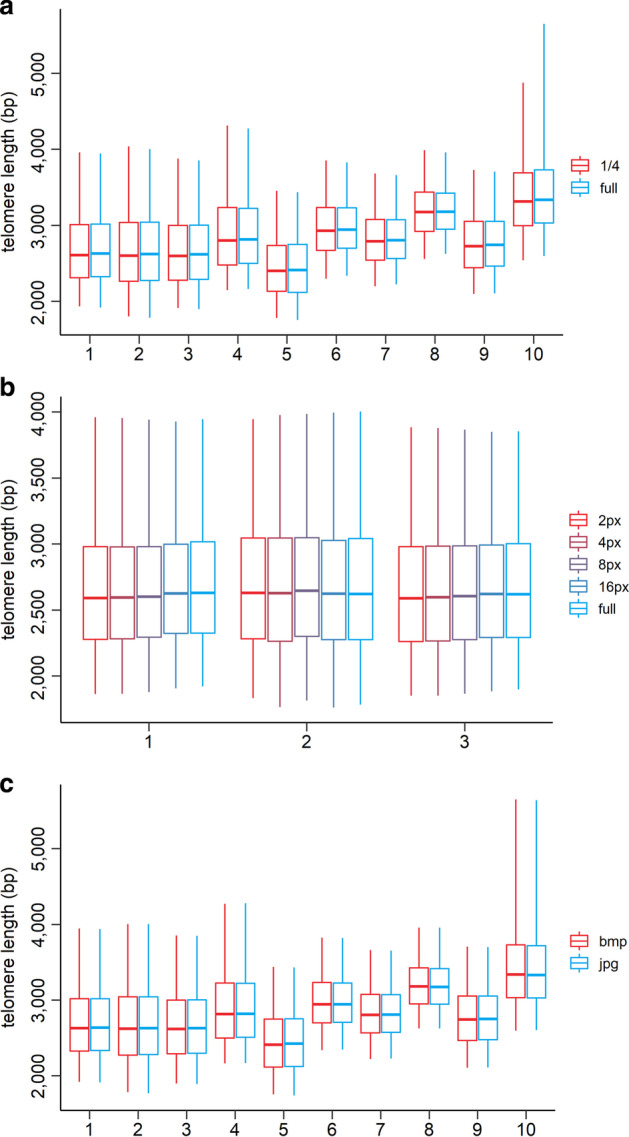
Impact of different properties of TRF scan and width of the area selection on the analysis by WALTER. (**a**) Influence of the scan size on the analysis. The full-size picture was 1644 × 1694 px, 1/4 size picture was 411 × 424 px. (**b**) Influence of the width of selected area of telomeric signal on the analysis. (**c**) Influence of the format of the file used for the analysis. Groups were compared by the “Overall visible spread” test (in the case of different widths, all groups were compared with the full width group). Every telomeric signal was measured 10 times and values were averaged. (Figure created using R 3.5.3)

A higher-resolution TRF scan implies a longer processing time to calculate the intensity profiles using the ScanToIntensity tool. However, a comparison of results obtained by evaluating a TRF scan with resolution 1644 × 1696 px and a version scaled to a fourth of its size revealed no significant difference in the outcome (Fig. 5a). Therefore, it is possible to scale down the resolution of TRF scans to allow a more rapid recalculation by the ScanToIntensity tool without introducing bias to the calculation of the distribution of intensity profiles. However, considering how it is calculated (see Implementation), the statistical evaluation will be slightly less sensitive with a lower resolution, so in cases when statistical evaluation of samples follows, analysis of a scan with the full resolution is recommended. Another issue that may come up during the calculation of intensity profiles and could affect the analysis of telomere lengths is the presence of blotches, stains, and other technical artefacts within the area of the telomere-specific signal. If possible, it is recommended to avoid areas of signals affected by these defects by selecting a narrower sector of the telomeric signal for the calculation of intensity profiles. As Fig. 5b shows, this manipulation does not affect the outcome of the analysis. Furthermore, the evaluation of TRF scan files of different formats yields the same results (Fig. 5c).

### Applicability of the WALTER toolset to broad spectrum of telomeres

**Fig. 6:**
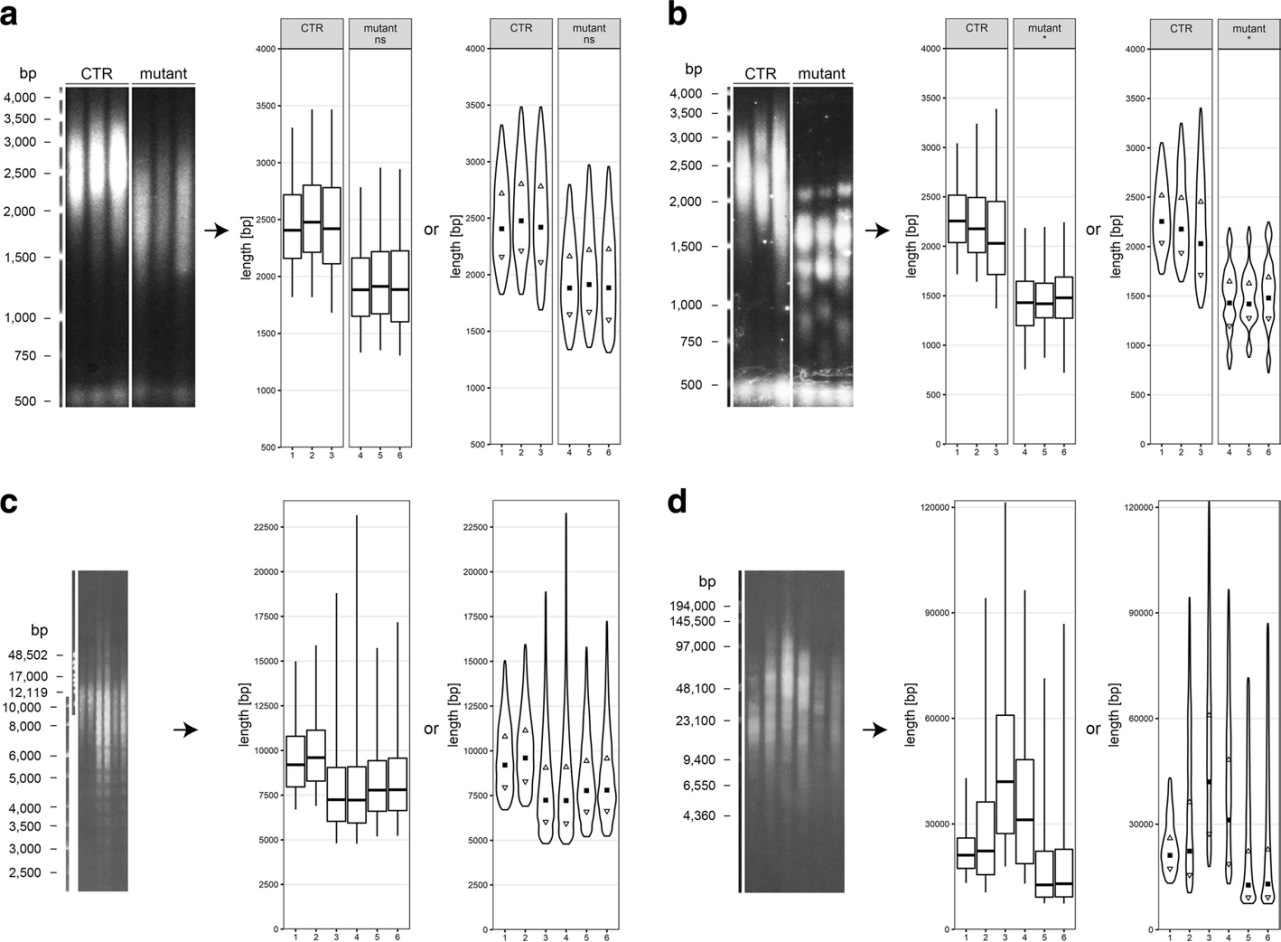
Applicability of the WALTER toolset to a broad spectrum of telomeres. Telomeres of *Arabidopsis thaliana* control and mutant plants **(a)**, *Arabidopsis thaliana* control plants and *tert* mutants with typical band-like pattern (**b**), human cells (**c**), and *Nicotiana* species (**d**) were analysed. In the boxplot, the central line indicates the weighted median, the box limits indicate the 25th and 75th percentiles and whiskers indicate the minimum and maximum of the selected area of telomeres. In the violin plot, the rectangle indicates the weighted median, triangles indicate the 25th and 75th percentiles and the shape of the violin plot depicts the intensity changes between minimum and maximum of the selected area of telomeres. (Figure created using Adobe Photoshop CS6)

To demonstrate the robustness of our toolset, we tested WALTER using TRF scans with telomeres differing significantly in their profiles or lengths (Fig. 6). We analysed relatively short telomeres in wild type plants *Arabidopsis thaliana* and mutants with a unimodal distribution of telomere-specific signal (Fig. 6a) as well as mutants with a fragmented telomere-specific signals (Fig. 6b). This pattern is also commonly present in human tumour cells that use the telomerase-independent mechanism for telomere maintenance—alternative lengthening of telomeres [11]. For evaluation of such complex types of TRF scans, no tool has been made available so far. Moreover, the nature of the specific distribution of the telomeric signal is reflected when using the option to present results as violin plots (Fig. 6b). In these two exemplary analyses, groups were also statistically compared using the option included in the online tool. We then analysed telomeres in human cells as representatives of medium-sized telomeres (Fig. 6c). With the WALTER toolset, it is also possible to analyse TRF profiles of long telomeres, as demonstrated for plant species of the genus *Nicotiana* (Fig. 6d).

### Comparison of WALTER with TeloTool

**Fig. 7:**
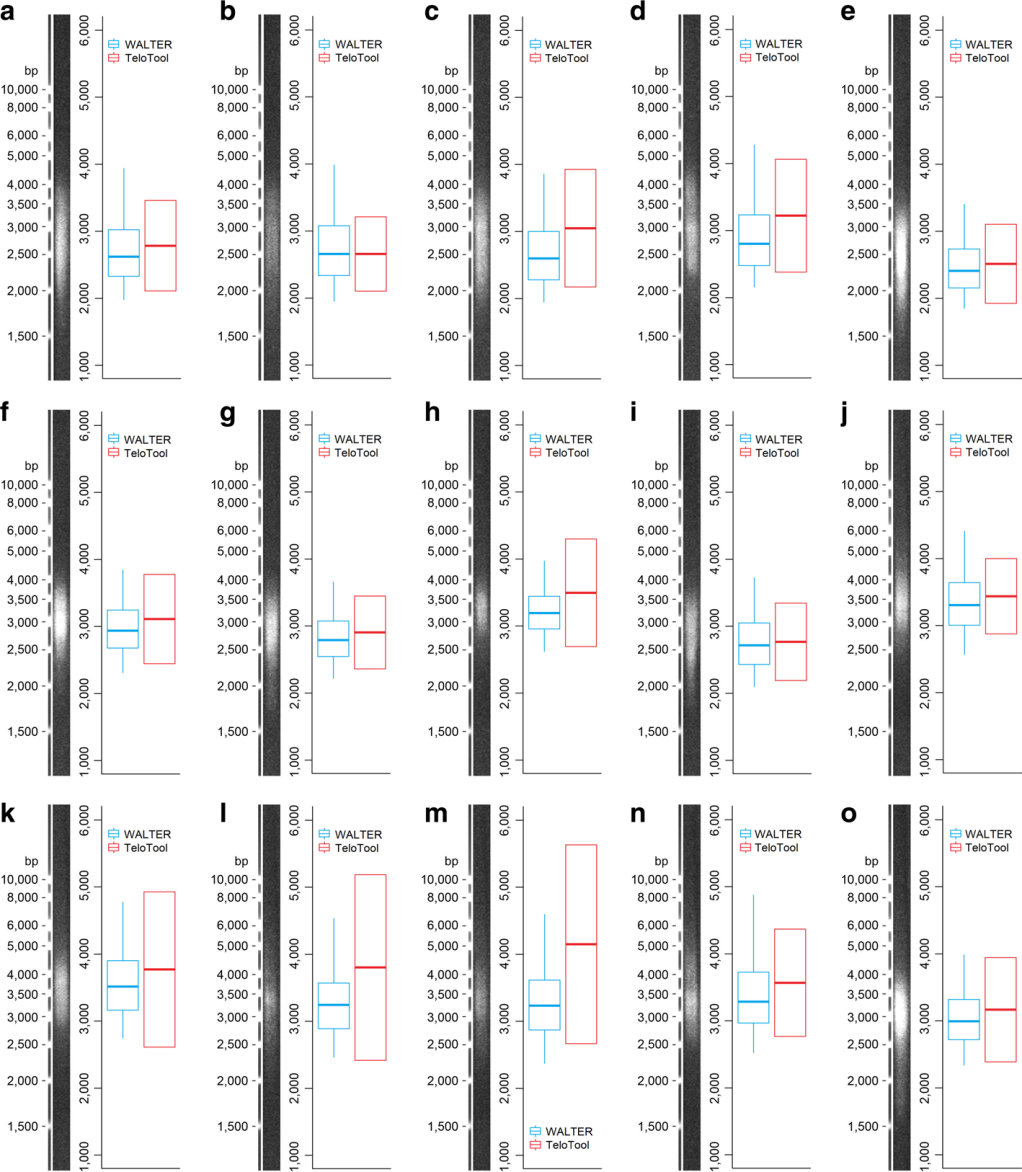
Comparison between WALTER and TeloTool. (**a-o**) Telomeric signals analysed by WALTER and TeloTool, respectively. In the case of the WALTER result, the central line indicates the weighted median, box limits indicate the 25th and 75th percentiles and whiskers indicate the minimum and maximum of the selected area of telomeres. In the case of TeloTool, the central line indicates the mean, and the box delimits the SD values; the corrected data option in TeloTool was used to obtain the values. (Figure created using Adobe Photoshop CS6 and R 3.5.3)

We compared the WALTER toolset to previously published TeloTool [7] that is considered to be the state-of-the-art used for the analysis of TRF scans. This tool uses a different approach for evaluating telomere-specific signals. While WALTER analyses smoothed raw telomeric signals (see Implementation), TeloTool fits the telomeric signal with a Gaussian function [7]. For this reason, utilization of the TeloTool is limited to TRF profiles with a unimodal distribution of the telomere-specific signal. Therefore, it is not applicable, e.g., for evaluating telomere lengths in *A. thaliana* mutants (Fig. 6b). A detailed comparison of both tools revealed that TeloTool tended to overestimate the length of telomeres (Fig. 7c, d, h, k–n), especially in those instances when TeloTool estimates were of low fit quality, which happened in our comparison 4 times out of 15 analysed samples (Fig. 7h, k–m).

## Discussion

The analysis of lengths of telomeres is an integral part of numerous studies, especially those dealing with the ecology and adaptation of organisms to environmental changes, human diseases, and ageing processes. Thus, the demand to precisely analyse results of TRF, still one of the most popular and reliable techniques for the telomere length analysis, is high. Although the TRF method itself is relatively simple, the evaluation of the data in a reproducible way represents the most complicated part of the analysis. Previous attempts to give the scientific community tools that would unify the way how TRF scans are analysed (Telometric [6] and TeloTool [7]) have not been widely adopted which led to the development of other manual techniques to interpret telomere lengths from the raw data [12–15]. However, these methods show a significant variation in data interpretation [15], not to mention that they also require a lot of time and effort to analyse a single membrane. Therefore, a user-friendly and robust tool possessing high quality, objective and reproducible evaluation of TRF data is of a high demand.

To answer this demand, we have developed a WALTER toolset that allows analysing a broad spectrum of TRF scans (Fig. 6). In comparison with TeloTool, our toolset allows analysis of scans with a non-unimodal distribution of the telomere-specific signal. This advantage is a result of how the TRF data is interpreted. While TeloTool is using a commendable approach to include the whole intensity profile in their analysis to fit the telomeric signal with a Gaussian function to get rid of the user bias [7], in our tool just a part of the telomere-specific signal from the intensity profile is interpreted. To get rid of the user bias, we set a threshold that automatically selects the appropriate area. Another benefit of our approach is that the analysed TRF scan can include technical defects of high signal intensity (e.g. stains, blotches, interstitial telomeric sequences) in the intensity profile outside of the telomere-specific signal while TeloTool would not be able to handle that. Also, given that ScanToIntensity tool relies on the manual selection of lanes, contrary to the automatic approach in TeloTool, our toolset allows the user to save intensity profiles of just partially selected lane (given that the other part might contain technical defects in the area with telomere-specific signal). The concern that partial selection of a lane might affect the calculation was refuted (Fig. 5b). Similarly, changes of other TRF scan parameters as the resolution of the scan or its format did not show any difference in the output of the analysis (Fig. 5a, c).

More importantly, we have shown that, in specific cases, the TeloTool overestimated the length of telomeres (Fig. 7c, d, h, k–n). This was prevalent especially when TeloTool estimated that its calculation is of low fit quality. Given that the TRF scan used for this comparison was of standard technical quality, it was rather surprising that this happened relatively frequently (4 times out of 15 analysed samples: Fig. 7h, k–m). As TeloTool provides the user with mean/SD values for each sample, it is possible to use this data for statistical evaluation of data. However, the low fit quality estimation by TeloTool that leads to the overestimation of telomere lengths is relatively frequent, thus it would require a quite high number of samples within each group to overcome this issue. But even then, the results would not be completely unbiased given that TeloTool overestimates results in some instances in general. On the other hand, WALTER toolset enables an in-house statistical evaluation of the data with the possible comparison between the control group and case groups or among all groups.

However, it should be noted that the illusive simplicity of the WALTER toolset in combination with the relative freedom provided to the user during the analysis might lead to a biased result when the best practice is not followed. Therefore, the user manual pinpointing critical steps, possible limitations and their troubleshooting needs to be read carefully. Even in this case, a presentation of input gel image and final log file represents the analysis transparently and reproducibly.

## Conclusions

WALTER is an R-based online toolset that fills a gap in telomere length analysis using telomere restriction fragment method that lacks commonly used tool. Its easiness to use, in-house statistical evaluation and applicability to wide variety of telomeres allow the scientific community to analyse TRF data in an unprecedented precise and reproducible way.

### Availability and requirements

Project name: WALTER; Project home page: https://www.ceitec.eu/chromatin-molecular-complexes-jiri-fajkus/rg51/tab?tabId=125#WALTER and https://github.com/mlyc93/WALTER; Operating system(s): Server-based version—platform independent; Portable version—Windows OS; Programming language: R; Other requirements: web browser, good quality internet connection; License: GNU Affero General Public License v3.0; Any restrictions to use by non-academics: Licence needed.

## Data Availability

WALTER toolset and user manual describing the evaluation of TRF scans in detail, including tips and troubleshooting, as well as, test data to demo the software can be accessed at https://www.ceitec.eu/chromatin-molecular-complexes-jiri-fajkus/rg51/tab?tabId=125#WALTER. There is also possible to download the portable version of the WALTER toolset. Source codes for ScanToIntensity and IntensityAnalyser can be obtained from https://github.com/mlyc93/WALTER. Biological material used in this study: *Arabidopsis thaliana*, ecotype Columbia and *Arabidopsis thaliana tert* mutant (SALK_061434), Nottingham Arabidopsis Stock Centre (UK); *Nicotiana tabacum* (acc. 095-55), Royal Botanic Gardens, Kew (UK); *Nicotiana sylvestris* (‘Seita’), Institut du Tabac, Bergerac (France); *Nicotiana rustica*, kind gift from Prof. Andrew Leitch (Queen Mary University London, UK); MSC (mesenchymal stem cells) and EPC (endothelial progenitor cells), 3H Biomedical, Uppsala (Sweden).

## Abbreviations

TRF: Terminal restriction fragment
WALTER: Web-based analyser of the length of telomeres

## References

[1] Montpetit AJ, Alhareeri AA, Montpetit M, Starkweather AR, Elmore LW, Filler K (2014). Telomere length: a review of methods for measurement. Nurs Res.

[2] Lee M, Napier CE, Yang SF, Arthur JW, Reddel RR, Pickett HA (2017). Comparative analysis of whole genome sequencing-based telomere length measurement techniques. Methods.

[3] Petracek ME, Lefebvre PA, Silflow CD, Berman J (1990). Chlamydomonas telomere sequences are A + T-rich but contain three consecutive G·C base pairs. Proc Natl Acad Sci USA.

[4] Fajkus J, Kovařík A, Královics R, Bezděk M (1995). Organization of telomeric and subtelomeric chromatin in the higher plant Nicotiana tabacum. Mol Gen Genet.

[5] Fojtova M, Fajkus P, Polanska P, Fajkus J (2015). Terminal restriction fragments (TRF) method to analyze telomere lengths. Bio-protocol.

[6] Grant JD, Broccoli D, Muquit M, Manion FJ, Tisdall J, Ochs MF (2001). Telometric: a tool providing simplified, reproducible measurements of telomeric DNA from constant field agarose gels. Biotechniques.

[7] Göhring J, Fulcher N, Jacak J, Riha K (2014). TeloTool: a new tool for telomere length measurement from terminal restriction fragment analysis with improved probe intensity correction. Nucleic Acids Res.

[8] Chang W, Cheng J, Allaire JJ, Xie Y and McPherson J. shiny: Web Application Framework for R. https://CRAN.R-project.org/package=shiny.

[9] Wan X, Wang W, Liu J, Tong T (2014). Estimating the sample mean and standard deviation from the sample size, median, range and/or interquartile range. BMC Med Res Methodol.

[10] Higgins JPT, Thomas J, Chandler J, Cumpston M, Li T, Page MJ, Welch VA (editors). Cochrane handbook for systematic reviews of interventions. 2nd ed. Wiley; 2019. 10.1002/9781119536604. 10.1002/14651858.ED000142 PMC10284251 31643080

[11] Bryan TM, Englezou A, Gupta J, Bacchetti S, Reddel RR (1995). Telomere elongation in immortal human cells without detectable telomerase activity. EMBO J.

[12] Kimura M, Stone RC, Hunt SC, Skurnick J, Lu X, Cao X (2010). Measurement of telomere length by the southern blot analysis of terminal restriction fragment lengths. Nat Protoc.

[13] Mender I, Shay J (2015). Telomere restriction fragment (TRF) analysis. Bio-protocol.

[14] Jenkins FJ, Kerr CM, Fouquerel E, Bovbjerg DH, Opresko PL (2017). Modified terminal restriction fragment analysis for quantifying telomere length using in-gel hybridization. J Vis Exp.

[15] Lincz LF, Scorgie FE, Garg MB, Gilbert J, Sakoff JA (2020). A simplified method to calculate telomere length from Southern blot images of terminal restriction fragment lengths. Biotechniques.

